# An Electrospun Fibrous Eye Mask with Antibacterial and Antioxidant Functions

**DOI:** 10.3390/biom16040554

**Published:** 2026-04-09

**Authors:** Xinhang Duan, Leting Wang, Chuxuan Cheng, Yili Zhang, Bingyue Guo, Hantong Wang, Jinghui Shi, Wenliang Song

**Affiliations:** 1 School of Materials and Chemistry, University of Shanghai for Science and Technology, Shanghai 200093, China; 2335052911@st.usst.edu.cn (X.D.); 254203044@st.usst.edu.cn (L.W.); 2 School of Artificial Intelligence Science and Technology, University of Shanghai for Science and Technology, Shanghai 200093, China; 2335053110@st.usst.edu.cn (C.C.); 2235053528@st.usst.edu.cn (Y.Z.); 2435062001@st.usst.edu.cn (B.G.); 2435061904@st.usst.edu.cn (H.W.); 3 Runfangke (Shanghai) Biotechnology Co., Ltd., Shanghai 201400, China; shijinghui@revacl.com

**Keywords:** electrospinning, nanofibers, eye mask, antibacterial, polylysine

## Abstract

Ocular infections and inflammation represent a clear risk to eye health, but standard eye masks often lack the necessary therapeutic features. Moreover, most existing studies employ a blended electrospinning approach, which leads to an inhomogeneous spatial distribution of the therapeutic agents. However, using the coaxial technique can address these limitations. This study develops a coaxial electrospun nanofibrous eye mask with dual antibacterial and antioxidant functions, aiming to provide an innovative ocular treatment tool for eye care. Generally, a core-shell structured bilayer polycaprolactone-polylysine/polyvinyl alcohol-resveratrol (PCL-PLs/PVA-RSV) membrane is successfully prepared by coaxial electrospinning, where the core is resveratrol-loaded PVA and the shell is PLs-loaded PCL. Results show uniform fiber morphology, favorable hydrophilicity, and potential for sustained release due to core-shell design. The membrane significantly inhibits the growth of *Staphylococcus aureus* (*S. aureus*) and *Escherichia coli* (*E. coli*); at the same time, it exhibits excellent free radical scavenging ability and good component biocompatibility, achieving slow release of the two drugs and long-term antioxidant effect. This multifunctional platform offers a synergistic approach to combating microbial infection and oxidative stress, showing great potential for eye care.

## 1. Introduction

Conventional eye masks are mainly designed to provide physical protection, cooling/heating effects, or simple moisturizing benefits; however, they generally lack active therapeutic functions for periocular care [1]. In particular, current eye-mask materials rarely integrate antibacterial and antioxidant activities, although the periocular region is highly susceptible to microbial contamination, oxidative stress, and irritation during prolonged wear [2,3,4]. Even when bioactive agents are incorporated, most existing systems rely on simple blending or surface loading strategies, which often result in limited loading stability, poorly controlled release behavior, and an inability to accommodate the distinct therapeutic time windows required for multiple active compounds [5]. Therefore, there remains a lack of functional eye-mask materials that can simultaneously achieve effective bioactive loading, structural stability during use, and temporally differentiated delivery of multiple agents [6].

Most currently available systems are unable to simultaneously provide effective antibacterial and antioxidant functions, and it remains particularly challenging to integrate these two activities within a single blend-electrospun fiber. This difficulty mainly arises because the two active components often differ in their physicochemical properties as well as in their required therapeutic time windows. In blend fibers, both agents are distributed within the same polymer matrix, which may lead to coupled or poorly controlled release and makes it difficult to assign distinct time-dependent roles to each component [7]. Electrospun nanofibrous membranes, by contrast, typically feature a high specific surface area and a tunable porous architecture, while their mechanical and wetting properties can be tailored through process parameters and material selection. In this context, a coaxial core-shell architecture offers a feasible solution by spatially separating the active agents into different compartments. Such a structure makes it possible for the shell layer to provide rapid antibacterial protection at the external interface during the initial stage of application, while simultaneously serving as a barrier that protects the antioxidant component encapsulated in the core from premature depletion and supports its more sustained release [8].

Resveratrol (RSV) is a naturally derived compound commonly extracted from plants such as grapes. It exhibits notable bioactivity and strong antioxidant as well as antibacterial capacities [9], and it has shown pronounced inhibitory effects against a broad range of bacteria and fungi. When used as a functional component in an eye mask, RSV may promote vasodilation and neuroprotection by activating endothelial nitric oxide synthase (eNOS) to generate nitric oxide. It may also suppress oxidative stress, reduce apoptosis, and modulate anti-inflammatory factors through activation of sirtuin 1 (SIRT1). Owing to its potent antioxidant activity, RSV can effectively scavenge free radicals and may therefore help protect blood vessels and nerves in the periocular region [10]. As a natural polyphenol, RSV is generally considered biocompatible; however, its safety for long-term periocular wear still requires further experimental evaluation. In addition, RSV may interfere with melanogenesis and thereby help reduce hyperpigmentation.

Polylysine (PLs), by contrast, is an efficient preservative and antibacterial agent. Its broad-spectrum antibacterial activity makes it suitable for addressing skin problems associated with bacterial infection [11]. In addition, hydrophilic groups such as amino moieties along the PLs chain may improve the wettability of the material, and PLs has also been reported to be potentially associated with inflammation-related processes [12]. Loading PLs into the shell layer can increase its interfacial availability and support early antibacterial action, whereas confining RSV in the core enables the shell to serve as a diffusion barrier that delays antioxidant depletion and facilitates its sustained delivery. Furthermore, because the PVA phase is water-soluble, a protective PCL shell can help maintain structural integrity during use, which is difficult to achieve with simple blended fibers.

Therefore, in this study, we proposed the concept of coaxial core-shell structure nanofibers, where PCL is used as the shell to load PLs and PVA is used as the core to encapsulate RSV. Through such spatial compartmentalization, temporal functional allocation can be achieved: shell-loaded PLs is expected to rapidly establish an antibacterial barrier at the initial stage of use, while core-encapsulated RSV, hindered by the shell diffusion barrier, may provide a more sustained release profile and prolonged antioxidant protection while maintaining cytocompatibility for periocular skin contact [13]. Meanwhile, to improve hygiene and sustainability, a replaceable-component concept can be adopted, in which the drug-loaded electrospun nanofibrous membrane serves as a disposable functional component, whereas the outer eye-mask holder is designed to be reusable. To validate the performance of the proposed nanofibrous membrane, we will systematically evaluate fiber morphology and the core-shell structure, release kinetics of the active agents, antibacterial activity, antioxidant-related metrics, as well as cytocompatibility.

## 2. Materials and Methods

### 2.1. Materials

The primary materials used in this experiment include PCL (M_w_ = 80,000 g·mol^−1^), PVA (degree of hydrolysis = 88–89%, M_w_ = 27,045 g·mol^−1^), and 2,2,2-Trifluoroethanol (TFE), which were purchased from Macklin Biochemical Co., Ltd. (Shanghai, China). RSV (purity ≥ 99.0%) was purchased from Tokyo Chemical Industry Co., Ltd. (TCI, Tokyo, Japan). Fluorescein (purity ≥ 95%) was purchased from Aladdin (Shanghai, China). L (+)-ascorbic acid (L-AA) (purity 99% (HPLC), CAS 50-81-7) was purchased from Adamas (Shanghai, China). A typical synthetic PLs (de-protection results in star-shaped poly-L-lysine, DP = 30, 1% *w/w* relative to PCL) method employs a ring-opening polymerization, the specific steps of which have been detailed in our previous publication [14].

### 2.2. Preparation of Spinning Solutions and Nanofiber Membranes

To prepare the shell spinning solution, 1 g of PCL is dissolved in 10 mL of TFE in a beaker. The mixture is stirred at 500 rpm at room temperature until the PCL is completely dissolved, yielding a 10% (*w/v*) homogeneous solution. Subsequently, 10 mg of PLs is added to this solution and stirred until well dispersed.

Simultaneously, the core spinning solution is prepared by dissolving 0.7 g of PVA in a mixed solvent of 5 mL deionized water and 5 mL ethanol. The mixture is heated at 80 °C under magnetic stirring at 500 rpm until a clear homogeneous solution is obtained, yielding a 7% (*w/v*) PVA solution. Then, 50 mg of RSV (7.1% *w/w* relative to PVA) is added to the solution and stirred until fully dissolved.

In order to ensure the effective release of PVA dissolved in water, a core-shell structured nanofiber membrane with a hydrophilic core and a hydrophobic shell was prepared. The solutions are loaded into separate syringes and mounted on micro-syringe pumps. A positive voltage of 8 kV is applied to the needle tip, while the aluminum foil-covered collector is grounded. The optimal flow rates are determined to be 0.2 mL/h for the PVA solution and 1.2 mL/h for the PCL solution. The distance between the tip and the collector is fixed at 15 cm. The electrospinning process is conducted at 25 °C and 40% relative humidity. Finally, the collected membranes are dried in a vacuum oven to remove residual solvents.

After the electrospinning process, the nanofiber membranes are collected and dried in a vacuum oven at room temperature for 24 h to remove residual solvents. Finally, the samples are stored in a desiccator for subsequent characterization.

### 2.3. Manual Cutting and Assembly of the Eye Mask

To enhance the sustainability of the device, a “replaceable insert” strategy is adopted. The electrospun drug-loaded fibrous membrane is independent of the outer shell of the eye mask, and the nanofiber membranes are cut into specific shapes tailored to the human periorbital contour. These shaped membranes are then inserted into the inner layer of a reusable eye-mask shell. This design allows the internal nanofiber layer to be stored under sealed conditions. When needed, it can be placed into the eye-mask shell to come into contact with the skin and exert its antibacterial and antioxidant properties. The shell can be reused and will not be contaminated due to contact with the skin. This approach ensures both hygiene and the efficacy of the active ingredients while significantly reducing material waste, aligning with the principles of green and sustainable development.

### 2.4. Characterization

The chemical structure of the fiber membranes is analyzed using FT-IR (SPECTRUM 100, PerkinElmer, Shelton, CT, USA) in Attenuated Total Reflectance (ATR) mode. The spectra are scanned in the wavenumber range of 4000–500 cm^−1^ with a resolution of 4 cm^−1^.

The micro-morphology of the fiber membranes is analyzed using a field emission SEM (Quanta FEG, FEI Company, Hillsboro, OR, USA) operated at an accelerating voltage of 10 kV. For SEM imaging, to enhance conductivity, the fiber membranes are first fixed onto aluminum stubs using double-sided conductive carbon tape, followed by sputter-coating with a thin layer of gold. The core-shell structure is further characterized by using a transmission electron microscope (Thermo Fisher Talos F200X G2, Waltham, MA, USA) at an accelerating voltage of 100 kV. The electrospun fibers are collected on carbon-coated copper grids for Transmission Electron Microscopy (TEM, Thermo Talos F200X G2, Thermo Fisher Scientific, Waltham, MA, USA) observation.

For wettability analysis, the fiber membranes are cut into 5 mm × 10 mm strips and placed on glass slides. The water contact angles are measured using a contact angle meter (DSA30, KRÜSS GmbH, Hamburg, Germany) equipped with a video capture system. In addition, fluorescence images of the fibers were acquired using a confocal laser scanning microscope (CLSM, ZEISS LSM 910, Carl Zeiss, Oberkochen, Germany) equipped with an Axio Imager.Z2 platform, image acquisition was performed using ZEN software (version 3.2, Carl Zeiss). And Scientific Compass (www.shiyanjia.com [M26.1][GR26.2]) performs the X-ray diffraction (XRD) analysis.

### 2.5. Antibacterial Activity

The antibacterial activity of the nanofiber membranes is evaluated using the plate counting method [15]. Two representative bacterial strains, *E. coli* and *S. aureus*, are selected. First, the bacterial strains are inoculated into LB medium and cultured overnight at 37 °C with continuous shaking at 200 rpm. The bacterial suspension concentration is then adjusted to 1 × 10^6^ CFU/mL. The experimental membrane with dimensions of 2 cm × 2 cm (PCL-PLs/PVA-RSV), the control membrane (pure PCL/PVA), Equal-content standard antibacterial agent quaternary ammonium salt (QA) and the blank group are incubated with 1 mL of the above bacterial suspension at 37 °C for 12 h. The culture mixtures are spread on agar plates, which are then incubated at 37 °C for an additional 12 h. Finally, the antibacterial efficacy of the nanofiber membranes is determined by counting the number of bacterial colonies formed on the agar plates. All experiments are performed in triplicate to exclude chance effects.

To further investigate the effect of the nanofiber membranes on cellular integrity, SEM imaging is used to analyze treated bacterial cells compared to control cells. The treated groups are prepared by co-culturing bacterial strains with sample membranes at 37 °C for 12 h, while an untreated bacterial group serves as the control. After incubation, bacterial cells are collected by centrifugation, washed with phosphate-buffered saline (PBS), and then fixed overnight at 4 °C in a 2.5% glutaraldehyde–paraformaldehyde solution. The fixed samples undergo dehydration through a graded ethanol series. After undergoing dehydration, the samples are subjected to freeze-drying. The surface morphology of the bacterial cells is subsequently examined by scanning electron microscopy (SEM).

### 2.6. Antioxidant Assay

The antioxidant activity of PCL-PLs/PVA-RSV nanofiber membranes was evaluated through the 1,1-diphenyl-2-propyl hydrazine radical scavenging test and the 2,2′-azobenzidine diphenylthiazol-6-sulfonic acid cation decolorization test. The PCL/PVA membranes were used as the negative control. The specific operation steps are as follows: Prepare a DPPH radical solution with a concentration of 0.3 millimoles. Prepare 40 mg of complete PCL-PLs/PVA-RSV nanofiber membranes and PCL/PVA nanofiber membranes, and add the free RSV of the same mass as that in the membranes and L-AA directly to approximately 5 mL of the aforementioned DPPH solution. In this experiment, the PCL/PVA nanofiber membranes were used as the negative control. The percentage antioxidant activity (%) is calculated using Formula (1) [16]:

(1) $$Scavenging\;Activity\;(\%)=(A_{B}-A_{S})/A_{B}\times 100$$

where *A_B_* is the absorbance of the PCL/PVA control and *A_S_* is the absorbance of the experimental sample measured by an Agilent Cary 60 UV-Vis spectrophotometer (Agilent Technologies, Santa Clara, CA, USA), distributed by Shanghai Jinghua Technology Instrument Co., Ltd., Shanghai, China. After a certain period of time when all the samples had clearly changed color, the images of the DPPH solution were collected to evaluate the color changes caused by the free radical scavenging activity of various experimental samples [17,18]. At the same time, ABTS cationic radical decolorization experiments are conducted. A 7 mM ABTS solution is reacted with a 2.45 mM potassium persulfate stock solution at room temperature in the dark for 12 h to generate ABTS cationic radicals. This solution is then diluted with ethanol before use to adjust its absorbance at 734 nm to 0.70 ± 0.02. Subsequently, the intact PCL-PLs/PVA-RSV nanofiber membranes, PCL/PVA nanofiber membranes, free resveratrol and L-AA are directly placed in approximately 5 mL of the above diluted ABTS solution [19,20]. After placing the sample in a dark environment, wait until a significant color change occurs, then measure the absorbance at a wavelength of 734 nm. Subsequently, calculate the ABTS free radical scavenging activity according to Formula (1).

### 2.7. Cell Culture and Cytocompatibility Evaluation

Cytotoxicity of the prepared polymers was evaluated by MTT assay using human immortalized keratinocyte (HaCaT) cells. HaCaT cells were seeded in 96-well microtiter plates and treated with various concentrations of polymers at 37 °C for 24 h. Subsequently, 100 μL of MTT solution was added to each well and incubated for an additional 4 h. The medium was then removed, and 200 μL of DMSO was added to dissolve the formed formazan crystals. Absorbance was measured using a microplate reader. Untreated HaCaT cells were used as the control.

### 2.8. In Vitro Release

To verify the release capacity of the nanofiber membrane, we prepared standard solutions containing RSV (0–50 μg/mL) and PLs (3–8 μg/mL). The absorbance was measured at wavelengths of 304 nm and 220 nm respectively, and a standard curve was plotted. Furthermore, in order to study the in vitro release behavior of PLs and RSV and to avoid possible interference during the experiments, each drug was loaded separately for individual release experiments. In the release test of PLs, only PLs was added to the outer layer, while the core layer did not contain RSV. In the release study of RSV, only RSV was added to the core layer, and the outer layer did not contain PLs. After preparing the samples, 30 mg of different nanofiber membranes were placed in dialysis bags (molecular weight cut-off value: 10 kDa), then immersed in 30 mL of PBS (pH = 5.4), and shaken at a speed of 80 revolutions per minute in a water bath at 37 °C for incubation. After a predetermined period of time, 4 mL of the release medium were taken out and replaced with an equal volume of fresh PBS. Then, the absorbance of the release medium was measured at wavelengths of 220 nm (for PLs) and 304 nm (for RSV). After obtaining the data, the cumulative release percentage was determined based on the standard curve, and the four kinetic models (zero-order model, first-order model, Korsmeyer–Peppas model, and Higuchi model) were evaluated to determine the rate of drug release.

## 3. Results

### 3.1. Preparation Strategy for the Eye Patch

**Figure 1:**
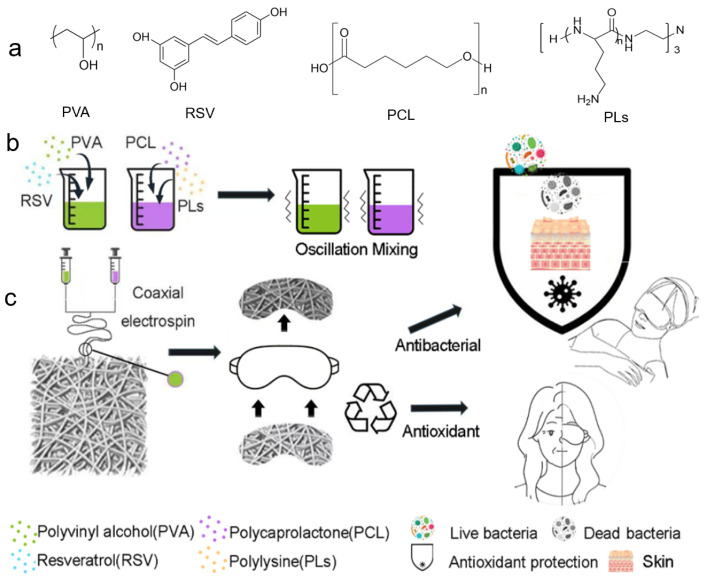
Coaxial electrospinning and core-shell nanofibers: (**a**) The structural formulas of PVA, RSV, PCL, and PLs. (**b**) The process of mixing PCL-PLs solution with PVA-RSV solution. (**c**) The concept design of eye patches based on core-shell nano-material films.

As shown in Figure 1, PVA is dissolved in an aqueous solvent containing RSV, and PCL is dissolved in TFE containing a specific amount of PLs, both under continuous magnetic stirring. In the coaxial electrospinning setup, a PVA solution serves as the core fluid, while a PCL solution is employed as the shell fluid [21]. The resulting core-shell structured nanofibers, designated as PCL-PLs/PVA-RSV, are fabricated using a custom-built electrospinning apparatus. For comparison, PCL/PVA coaxial core-shell nanofiber membranes, which do not exclude RSV and PLs are also prepared as control samples [22].

### 3.2. Characterization of the Fiber Membrane for the Eye Patch

**Figure 2:**
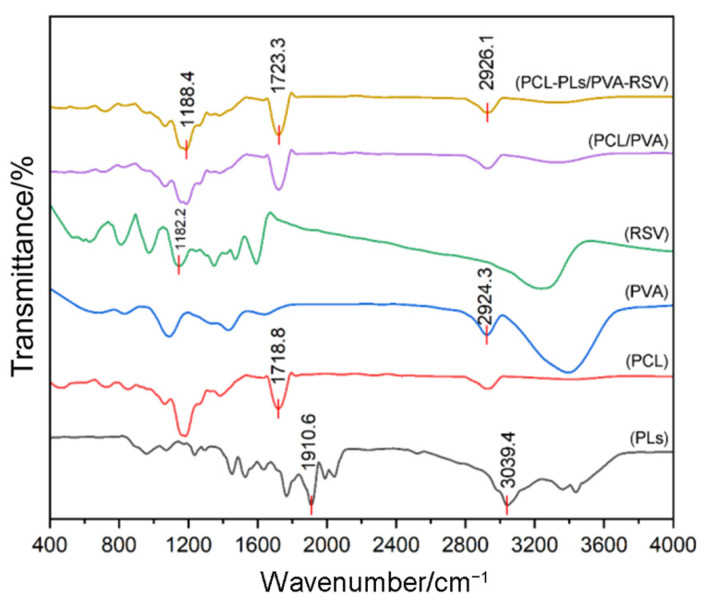
The FT-IR spectra of the nanofiber membrane.

FT-IR spectroscopy is performed to confirm the successful preparation of the PCL-PLs/PVA-RSV membrane [23]. As shown in Figure 2, in the spectrum of PCL-PLs/PVA-RSV, the characteristic peak at 1188.4 cm^−1^ is attributed to PVA respectively, confirming the successful loading of PVA. The characteristic peak at 2926.1 cm^−1^ originates from the aliphatic C-H stretching of PCL [24]. The original PLs exhibits characteristic peaks at 1910.6 cm^−1^ and 3039.4 cm^−1^ [25]; however, due to the low content of RSV and PLs, their characteristics are not reflected in FT-IR spectra.

**Figure 3:**
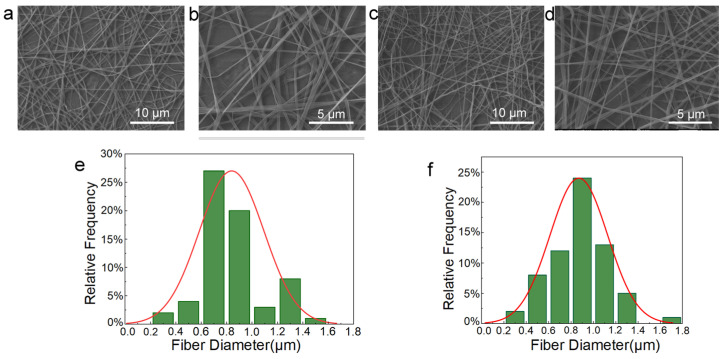
The fiber showing the SEM images and fiber diameter distributions of the fiber membranes (**a**,**b**,**e**) PCL-PLs/PVA-RSV, (**c**,**d**,**f**) PCL/PVA.

Figure 3a,b display SEM images of the PCL-PLs/PVA-RSV nanofiber membrane, showing a non-woven mat composed of randomly oriented fibers that intersect and bond to form a continuous three-dimensional porous architecture, with the average fiber diameter measured at around 870 nm. Figure 3c,d show SEM images of the PCL/PVA nanofiber membrane, with an average fiber diameter of approximately 840 nm. However, due to voltage instability during the spinning process, some fibers exhibit uneven diameters (Figure 3e,f).

**Figure 4:**
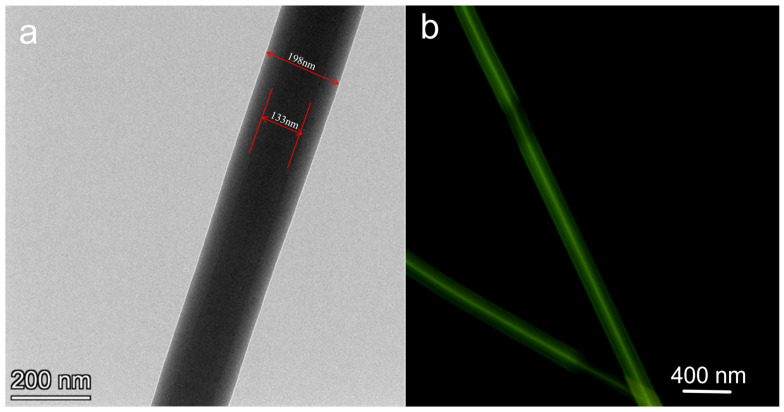
(**a**) The fiber showing the TEM images of PCL-PLs/PVA-RSV by coaxial electrospinning methods. (**b**) Fluorescence micrograph of PCL-PLs/PVA-RSV coaxial nanofiber membranes (fluorescein-labeled core layer).

To further verify the successful preparation of the PCL-PLs/PVA-RSV coaxial fiber membrane, TEM is utilized for characterization. TEM observations reveal distinct color differentiation between the core and shell regions of the fibers. TEM images further confirm the bilayer core-shell structure (Figure 4a). These results clearly indicate the successful formation of PCL-PLs/PVA-RSV coaxial nanofibers. In the fluorescence micrograph (Figure 4b), the fibers show continuous long green signals. The fluorescence is mainly concentrated within the fibers and forms a relatively continuous “bright core” along the axial direction, while the edges of the fibers are relatively dark, indicating that the fluorophore is mainly embedded in the PVA-RSV core layer and does not significantly migrate to the PCL-PLs shell layer, thereby supporting the formation of the coaxial core-shell structure. At the same time, there are certain fluctuations in brightness, which may be related to local fluctuations in the core layer diameter, interface diffusion/compatibility differences, or imaging defocus, and the stronger fluorescence at the overlapping areas should be avoided in quantitative analysis due to the thickness superposition.

PVA and PCL are two key polymers in biomaterial applications. PVA is a water-soluble synthetic polymer characterized by its excellent film-forming ability and non-toxicity. In contrast, PCL, a biodegradable polyester, is esteemed for its prolonged degradation period, high mechanical strength, and superior elasticity.

**Figure 5:**
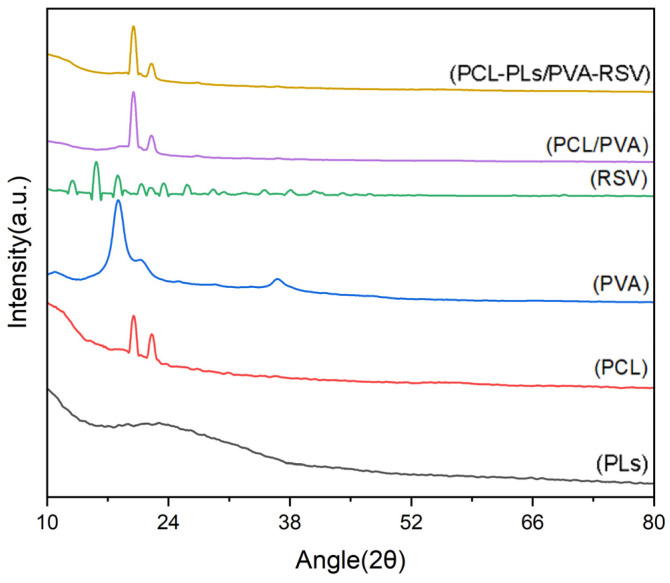
The X-ray diffraction of PLs, PCL, PVA, RSV and PCL/PVA.

Characterization is performed using TEM. The corresponding optical microscopy images indicate a well-defined color contrast between the inner and outer layers of the fibers. The pattern for PVA (Figure 5) is characterized by a broad halo centered at approximately 2θ = 19.5°, confirming its amorphous nature. In contrast, the pattern for PCL exhibits two sharp diffraction peaks at 2θ = 21.3° and 23.6°, which are assigned to the (110) and (200) crystal planes of PCL (ICDD PDF No. 00-060-0295), respectively, verifying its semi-crystalline structure [26,27,28]. Analysis of the PCL/PVA composite reveals that the characteristic peaks of PCL are preserved, indicating the retention of the PCL crystalline phase [23,24,25,26,27,28]. However, a significant reduction in the intensity of these peaks is observed, indicating a decrease in the overall crystallinity of the composite. This phenomenon is likely due to intermolecular interactions between PVA and PCL chains at their interface, which disrupt the ordered packing of PCL chains and inhibit crystal formation [24,26]. However, some spectra show unclear peaks, which may be due to the small sample size during the experiment.

A critical observation is the absence of distinct diffraction peaks corresponding to the loaded drug, PLs, in the composite’s spectrum. This strongly suggests that PLs is not present in a crystalline form but exists in a molecular or amorphous state, effectively encapsulated within the PCL/PVA fibrous matrix [29,30].

In summary, the XRD results confirm the successful formation of a composite material where the PCL crystalline phase is maintained but with changed crystallinity due to interfacial interactions with PVA. Meanwhile, the effective amorphous encapsulation of the PLs within the composite framework is demonstrated.

**Figure 6:**
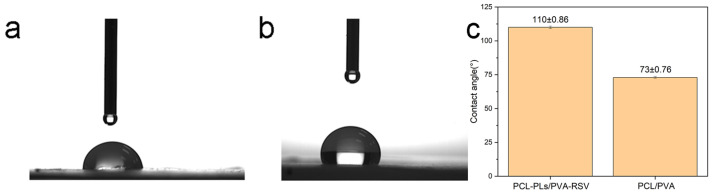
Contact angle images of (**a**) PCL-PLs/PVA-RSV, (**b**) PCL/PVA. (**c**) Contact angle bar chart of PCL-PLs/PVA-RSV and PCL/PVA nanofiber membranes. The data are presented as the means ± SDs (n = 3).

A fundamental function of eye patches is to moisturize the eye area. To verify the hydrophilic performance of the PCL-PLs/PVA-RSV fiber membrane, water contact angle tests are conducted on both PCL-PLs/PVA-RSV and PCL/PVA fiber membranes. The results are shown in Figure 6. The water contact angle of the PCL-PLs/PVA-RSV fiber membrane is clearly smaller than that of the PCL/PVA fiber membrane, confirming the improved hydrophilicity of the PCL-PLs/PVA-RSV fiber membrane.

### 3.3. Antimicrobial and Antioxidant Study

Most existing traditional eye masks only add basic care ingredients such as aromatic compounds and lutein, and generally lack core functions like antibacterial and antioxidant properties, making it difficult to cope with skin damage caused by periorbital microflora imbalance and oxidative stress. The PCL-PLs/PVA-RSV nanofiber eye mask prepared in this study achieves functional upgrading through innovative design.

The antibacterial and antioxidant functionalities of the nanofiber membrane depend on the PLs loaded in the PCL shell and the RSV loaded in the PVA core, respectively, working synergistically to protect the periorbital skin. The antibacterial mechanism of PLs originates from the cationic ammonium group (−NH^3+^) in its molecular structure: Upon hydration of the eye patch, the PCL shell slowly releases PLs, whose cationic groups can electrostatically interact with the negatively charged phospholipids on the bacterial cell membrane, disrupting its integrity. This leads to leakage of intracellular components such as proteins and nucleic acids, ultimately inhibiting bacterial growth or causing cell death. This effect is active against both Gram-positive bacteria (e.g., *S. aureus*) and Gram-negative bacteria (e.g., *E. coli*), demonstrating broad-spectrum antibacterial properties. The antioxidant function of core-loaded RSV is based on its phenolic hydroxyl structure, which can react with reactive oxygen species (ROS) generated by skin cell metabolism, neutralizing the oxidative activity of ROS and reducing oxidative stress damage to the sensitive periorbital skin. It might also assist in regulating the activity of the skin’s own antioxidant enzyme system, further enhancing the skin’s antioxidant capacity [23,31].

**Figure 7:**
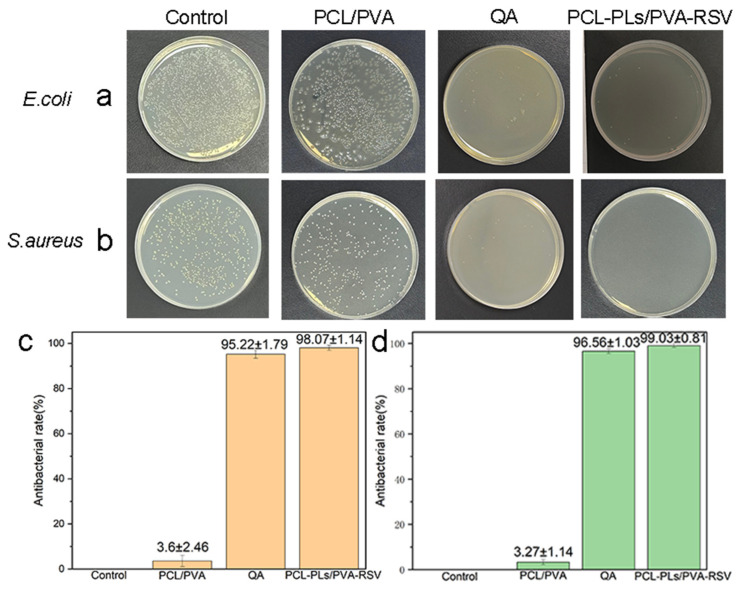
Photos of the colonies of (**a**) *E. coli* and (**b**) *S. aureus* on the blank control group and the agar plates separately added with PCL/PVA, QA and PCL-PLs/PVA-RSV fiber membranes. (**c**) The *E. coli* antibacterial rate and (**d**) *S. aureus* antibacterial rate of PCL/PVA nanofiber membranes, QA and PCL-PLs/PVA-RSV nanofiber membranes. The data are presented as the means ± SDs (n = 3).

To verify the antibacterial effect of the PCL-PLs/PVA-RSV fiber membranes, this study sets up a blank control group (blank control), a PCL/PVA fiber membrane control group, an PCL-PLs/PVA-RSV experimental group and a standard antibacterial agent QA positive control group. Cultivation experiments are conducted for *E. coli* and *S. aureus*. As shown in Figure 7, the Petri dishes of the blank control group are covered with dense colonies, indicating the good growth vitality of the strains. The diluted PCL/PVA control membranes without drug show no significant inhibitory effect on both *E. coli* and *S. aureus*: the number of colonies on the *E. coli* Petri dish is approximately 1000, and the number of colonies on the *S. aureus* Petri dish is approximately 350. This indicates that the pure polymer membranes themselves do not have antibacterial activity. The number of colonies on the *E. coli* culture plates in the QA positive control group was approximately 11–15, while the number of colonies on the *S. aureus* culture plates was approximately 5–10. However, after coculturing with the PCL-PLs/PVA-RSV composite membrane, the number of colonies on the Petri dishes decreases significantly: only 1–3 colonies remain on the *E. coli* Petri dish (average 10), and only 1–2 colonies remain on the *S. aureus* Petri dish (average 1). According to the antibacterial rate calculation formula, the standard antibacterial agent QA has an antibacterial efficacy of 95% against *E. coli* and 96% against *S. aureus*. The antibacterial rate of the PCL-PLs/PVA-RSV fiber membranes against *E. coli* is 98%, and against *S. aureus* is 99%, which is slightly higher than that of QA. Although there are still a few remaining colonies, the antibacterial effect is significant [13].

**Figure 8:**
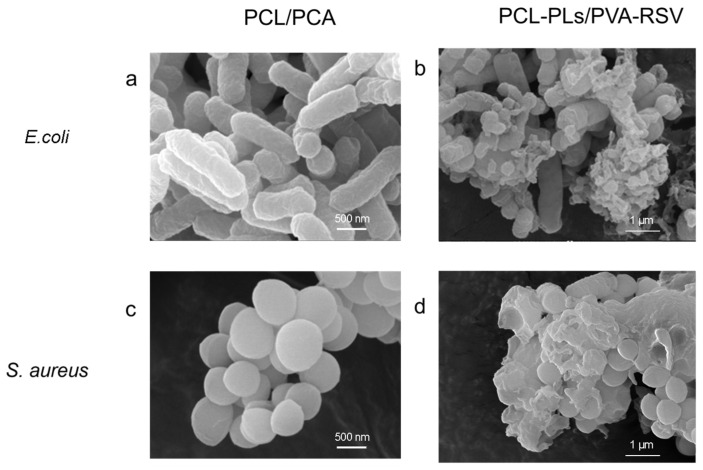
Photos of bacterial culture plates of (**a**,**b**) *E. coli* and (**c**,**d**) *S. aureus* treated with PCL/PVA and PCL-PLs/PVA-RSV fiber membranes.

To further elucidate the antibacterial mechanism of PLs, the morphological changes in bacteria after interaction with the fiber membranes are observed by SEM, focusing on comparing the structural differences in bacteria on different membrane surfaces (Figure 8). When not exposed to antibacterial components, the natural morphology of the bacteria exhibits distinct characteristics: *E. coli* appears rod-shaped, and *S. aureus* appears spherical. The cell membranes of both remain smooth and intact, with a plump structure and clear contours, which is typical for bacteria maintaining normal metabolic activity [32].

However, after contact with the PCL-PLs/PVA-RSV membrane, the bacterial morphology is significantly compromised: both bacterial types exhibit noticeable shrinkage and deformation. Their originally smooth cell membranes become rough and lose structural integrity, with some cells even developing ruptured pores. The observed morphological distortion and pore formation (Figure 8) are consistent with the well-documented antibacterial mechanism of PLs. It is hypothesized that the cationic ammonium groups (−NH^3+^) in its molecular structure interact electrostatically with the negatively charged phospholipids on the bacterial cell membrane [33]. This interaction likely destabilizes the membrane structure, leading to the potential leakage of critical intracellular components, such as proteins and nucleic acids, and ultimately resulting in the loss of normal physiological functions [34,35].

In stark contrast, bacteria on the PCL/PVA control membrane maintain their intact morphology, consistent with the natural state: the cell membranes remain smooth and undamaged, and the cell structures are plump, indicating unaffected metabolic activity. This result not only further confirms that PLs exerts its antibacterial effect by disrupting the bacterial cell membrane but also rules out interference from other components. It is particularly important to note that RSV, loaded in the shell layer of the membrane, does not exhibit antibacterial activity in this process. The two components operate without overlapping and synergistically protect the periorbital skin.

Furthermore, this correlation between “morphological damage” and “antibacterial effect” is consistently observed in both *E. coli* and *S. aureus*, demonstrating that the antibacterial mechanism of PLs is universal against these bacteria. This further highlights its broad-spectrum antibacterial properties, providing reliable protection for the periorbital skin against various bacterial infections [36].

**Figure 9:**
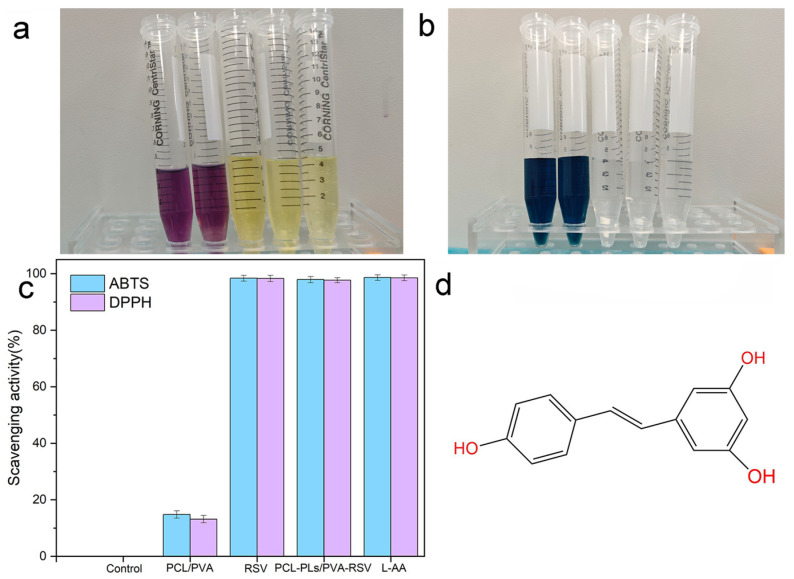
(**a**) PCL/PVA nanofiber membranes, free RSV, PCL-PLs/PVA-RSV nanofiber membranes, and L-AA treatment, the color change in the DPPH solution; (**b**) PCL/PVA nanofiber membranes, free RSV, PCL-PLs/PVA-RSV nanofiber membranes, and after L-AA treatment, the color change in the ABTS solution; (**c**) the ABTS and DPPH free radical scavenging activities of the control group, PCL/PVA nanofiber membranes, free resveratrol, PCL-PLs/PVA-RSV nanofiber membranes and L-AA; (**d**) chemical structure diagram of RSV.

To verify the antioxidant capacity of PCL-PLs/PVA-RSV, the antioxidant activities of PCL-PLs/PVA-RSV and PCL/PVA fiber membranes are assessed using DPPH radical scavenging and ABTS radical cation scavenging assays, and the free RSV and L-AA are used as a control for the assays. Figure 9 displays the results of the antioxidant activity evaluation for PCL-PLs/PVA-RSV, PCL/PVA fiber membranes, free RSV and L-AA based on DPPH and ABTS radical scavenging capacities. In this study, neither the control group nor the PCL/PVA nanofiber membrane induces solution decolorization, whereas PCL-PLs/PVA-RSV, free RSV and L-AA cause pronounced decolorization. The DPPH radical scavenging assay reveals a scavenging rate of 98.02 ± 1.09% for the PCL-PLs/PVA-RSV group, compared to only 13.1 ± 1.57% for the PCL/PVA group, while the clearance rate of the free RSV group was 98.97 ± 0.95%, and the clearance rate of the L-AA group was 99.08 ± 1.03%. In the ABTS radical scavenging assay, the scavenging rates were 97.92 ± 1.37% and 14.81 ± 1.58% for the PCL-PLs/PVA-RSV and PCL/PVA groups, the clearance rate of the free RSV group was 99.06 ± 1.24%, and the clearance rate of the L-AA group was 99.15 ± 1.08% respectively. As L-AA possesses excellent immediate antioxidant capacity, the clearance rates in the test were all higher than those of free RSV and PCL-PLs/PVA-RSV nanofiber membranes. The clearance rate of PCL-PLs/PVA-RSV was slightly lower than that of free RSV and L-AA, this might be related to two factors: Firstly, after RSV was loaded/embedded, some of its active sites were difficult to fully expose within the short reaction window, resulting in a slight decrease in the apparent clearance rate. Secondly, the mass transfer process of the fibrous membrane (free radicals diffusing into the membrane, RSV being released/migrated to the reaction interface from the carrier) would to some extent limit the instantaneous reaction efficiency.

However, this “slight decline” is not a drawback from an application perspective, indicating that the material has sustained antioxidant capabilities, which is beneficial for maintaining a longer-lasting antioxidant microenvironment during actual application. Meanwhile, the functional adaptability demonstrated by the composite nanofiber membrane under the existing experimental conditions can be inferred to provide a certain theoretical basis and practical foundation for its application as an antioxidant-active eye patch material in the field of skin dressings. It should be noted that the DPPH/ABTS test results of this measurement represent the final assessment of the membrane’s antioxidant capacity within the fixed reaction time. The continuous antioxidant performance during the process is inferred through the measured RSV release kinetics (Section 3.5), and this kinetic result determines the time-dependent availability of RSV in the surrounding medium.

**Figure 10:**
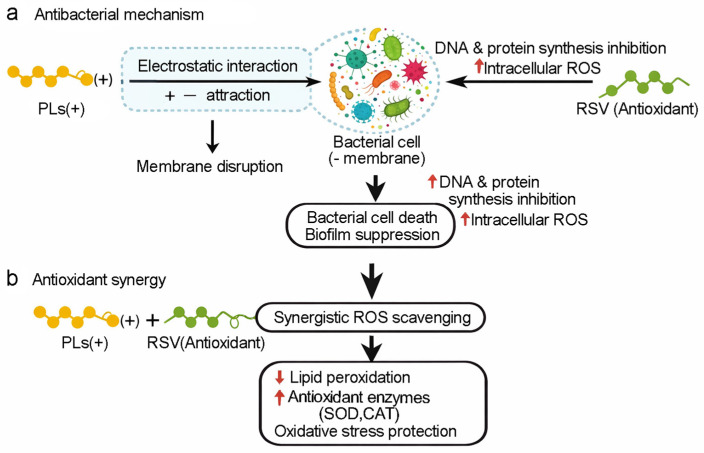
(**a**) Schematic diagram of the synergistic antibacterial mechanism, and (**b**) synergistic antioxidant mechanism of PLs and RSV.

Regarding the synergistic effect between PLs and RSV, as shown in Figure 10, PLs can cause the bacterial cell membrane to rupture and increase its permeability, while RSV can inhibit the synthesis of bacterial DNA and proteins and increase the level of ROS, thereby jointly promoting bacterial death. PLs can also work together with RSV to form enhanced ROS scavenging, thereby enhancing the antioxidant capacity.

Simultaneously, RSV, as a highly efficient natural antioxidant, shows significant value in combating skin aging. The core mechanism of skin aging stems from persistent oxidative stress. Environmental factors such as ultraviolet (UV) radiation can induce the production of ROS, which leads to eye inflammation reactions, skin aging and infection problems. Traditional cream formulations are plagued by issues like low bioavailability and poor stability. In contrast, the PCL-PLs/PVA-RSV core-shell structured nanofiber membrane prepared in this study serves as an advanced active ingredient delivery system. Coupled with its antibacterial properties and antioxidant activity, this nanofiber membrane holds promising potential for applications in eye patches and other skin dressing fields.

The aforementioned experimental results demonstrate that the PCL-PLs/PVA-RSV nanofiber membranes exhibit comprehensive antioxidant activity in fibrous form. As the primary interface between the human body and the external environment, the skin is highly susceptible to oxidative stress. Owing to their potent free radical scavenging capacity, these nanofiber membranes can effectively neutralize reactive oxygen species generated endogenously in cutaneous tissues, thereby safeguarding keratinocytes, structural proteins (e.g., collagen and elastin), and genomic DNA from oxidative damage—ultimately fulfilling a critical role in biological barrier protection.

### 3.4. Cell Culture and Cell Compatibility Results

**Figure 11:**
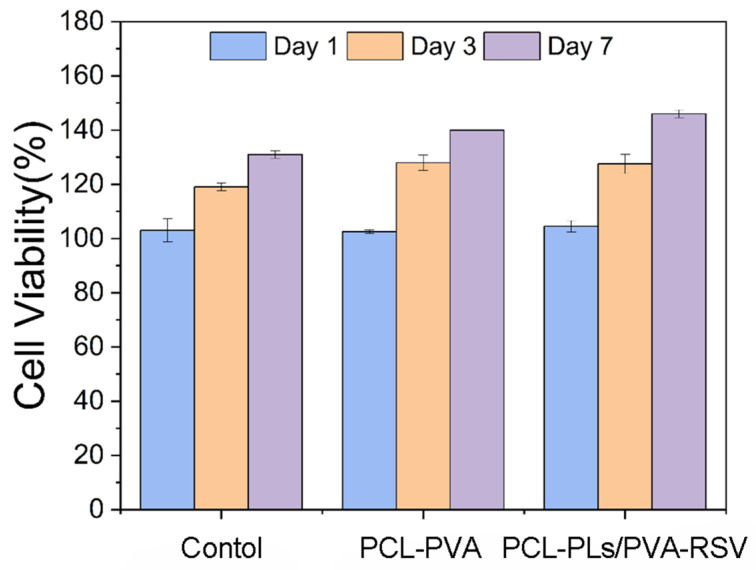
Cell viability of control, PCL/PVA, and PCL-PLs/PVA-RSV groups after 1, 3, and 7 days of incubation.

The cytotoxicity test was performed in accordance with ISO 10993-5:2023 [37]. The cell viability results (Figure 11) demonstrate that all samples maintained values above 100% throughout the incubation period, indicating the absence of cytotoxicity. Notably, the PCL-PLs/PVA-RSV group exhibited the highest proliferation rate at Day 7, suggesting that the composite scaffold provides a favorable microenvironment for cell attachment and growth. The progressive increase in viability over time further confirms the good biocompatibility of the developed materials. According to ISO 10993-5 standards, materials with cell viability above 80% are considered non-cytotoxic, All experimental groups significantly exceeded this threshold.

### 3.5. Release Behavior of PLs and RSV

To verify whether the “spatial separation–functional zoning” strategy of coaxial electrospinning can achieve differentiated release of PLs and RSV, this study focused on a dual-loaded coaxial composite fiber membrane as the research object, and used ultraviolet-visible spectrophotometry to separately determine the release behaviors of PLs and RSV. It should be noted that the coexistence of the two components in the combined system may affect the swelling and diffusion process of the membrane. Therefore, the “separate release” results are mainly used as an alternative indicator for comparing the differences in drug release, and are used to evaluate the effectiveness of the zoning design. The observed asynchronous release pattern provides indirect, design-consistent evidence supporting the core-shell architecture hypothesis.

**Figure 12:**
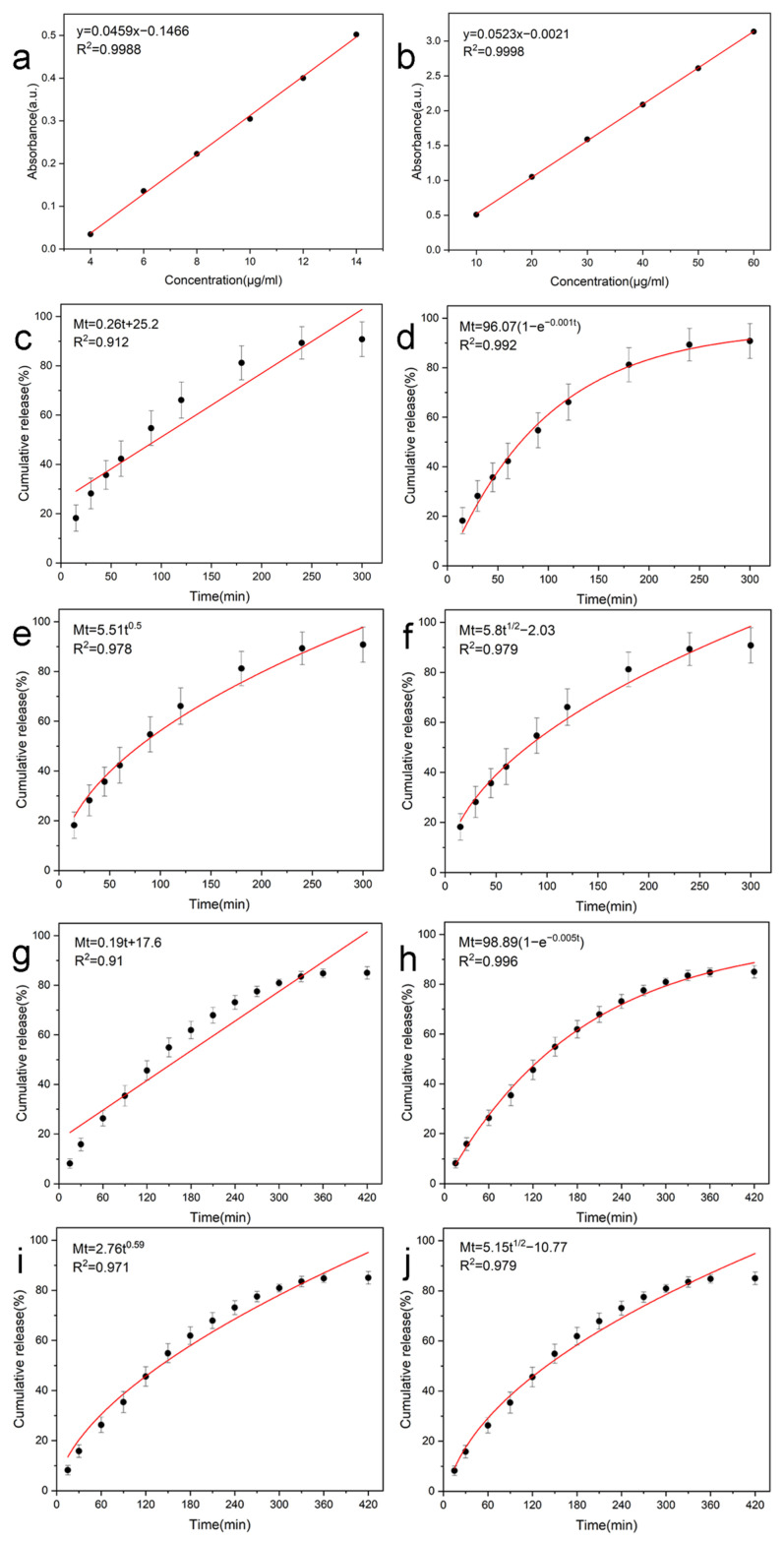
UV standard curves of (**a**) PLs and (**b**) RSV; the PLs release curves fitted by the four kinetic models are shown in (**c**) zero-order model fitting, (**d**) first-order model fitting, (**e**) Korsmeyer–Peppas model fitting, and (**f**) Higuchi model fitting; and the RSV release curves filled by (**g**) zero-order model fitting, (**h**) first-order model fitting, (**i**) Korsmeyer–Peppas model fitting, and (**j**) Higuchi model fitting.

This study verified the release kinetics of PCL-PLs/PVA-RSV nanofiber membranes using ultraviolet-visible spectroscopy. Firstly, a standard curve for PLs and RSV was established (Figure 12a,b). Both PLs (y = 0.0459x − 0.1466, R^2^ = 0.9988) and RSV (y = 0.0523x − 0.021, R^2^ = 0.9998) showed excellent linear relationships within the test range, laying the foundation for quantitative analysis.

As shown in Figure 12, both PLs and RSV exhibited typical three-stage release characteristics of drug-loaded nanofiber membranes. Taking RSV as an example (Figure 12g), a significant burst release effect was observed in the initial release stage (the first 60–120 min), which was attributed to the rapid dissolution of the drugs on the fiber surface into the medium; subsequently, the release curve entered a gradual sustained release stage (Sustained release), reflecting the slow diffusion of the drugs encapsulated in the fiber interior as the polymer matrix swelled and the chain segments relaxed; finally, the release rate gradually decreased and approached a plateau (Plateau). To determine the actual delivery dose of the system, this study additionally measured the loading/encapsulation efficiency (EE%) and drug loading content (DLC) of the two drugs in the core-shell nanofiber membranes. At 300 min and 420 min respectively, the drug content of the fiber membrane was quantitatively analyzed. The results showed that the drug loading efficiencies of PLs and RSV were 85.35% and 88.97% respectively, and the corresponding drug loading amounts were 8.54 μg/mg and 19.57 μg/mg. Subsequent experiments revealed that the drug release rate showed no significant improvement. These data provided a “dose benchmark” for the release behavior, thereby avoiding the problem that the release curve alone cannot be used to infer the actual delivery dose. In this experimental release system, the cumulative drug release amounts of PLs and RSV reached 7.25 μg/mL and 16.05 μg/mL, this is in line with the design of this coaxial nanofiber membrane, which is capable of achieving effective drug release under in vitro conditions.

Since RSV is the sole antioxidant component in this system, the RSV concentration measured in the release medium can be regarded as a function of the available antioxidant dose over time. Therefore, the initial burst, sustained diffusion, and plateau phase of RSV correspond to the process of antioxidant capacity being established rapidly in the early stage and then maintained subsequently. This is consistent with the high DPPH/ABTS scavenging rate exhibited by the RSV-containing membrane in Section 3.3, indicating that the released RSV can effectively scavenge free radicals within the measurement time window.

In this study, the membrane is composed of nanofiber spinning. For the convenience of mechanism analysis, it can be approximately regarded as a cylindrical geometric structure, and multiple kinetic models were fitted and analyzed (Figure 12c–j). The first-order kinetic model was used to fit the complete release curve, achieving the best overall fitting effect (with the highest coefficient of determination R^2^), This is consistent with the design of drugs where the overall release rate is mainly determined by the concentration gradient, that is, the drug release rate is related to the remaining releasable drug amount in the system, and presents a gradually decaying characteristic over time.

The Korsmeyer–Peppas model was used to fit the drug release indices of PLs and RSV, which were 0.50 (coefficient of determination R^2^ = 0.978) and 0.59 (coefficient of determination R^2^ = 0.971) respectively (Figure 12e,i). The values were all within the range of 0.45 to 0.89, indicating that the diffusion of both drugs is jointly governed by polymer-chain relaxation and swelling, as well as dissolution. It is important to note that the first-order kinetics law does not conflict with the non-Fickian diffusion mechanism proposed by Korsmeyer–Peppas: the first-order kinetic law is used to describe the macroscopic rate law of overall drug release (dominated by concentration gradients), while the n value of the Korsmeyer–Peppas model can reveal the contribution ratio of the diffusion–relaxation coupling mechanism during the drug release process (especially in the initial stage of drug release). In summary, the release characteristics of this system conform to the first-order kinetic decay law at the macroscopic level, and at the mechanistic level, it achieves the coordinated regulation of drug sustained-release diffusion and the evolution of the matrix structure.

This sequential release pattern is closely related to its coaxial structure: PLs located in the hydrophobic PCL shell can be rapidly released to provide immediate antibacterial effects, while RSV located in the hydrophilic PVA core layer achieves more sustained antioxidant protection under the dual effects of shell obstruction and core-controlled release. The comprehensive research results indicate that the PCL-PLs/PVA-RSV nanofiber membranes can achieve asynchronous dual-drug release—the rapid release of PLs provides immediate antibacterial protection during the early wearing stage, while the continuous controlled release of RSV maintains continuous antioxidant activity around the eye throughout the entire usage period, and can be applied to sleep masks for 8 h overnight use needs. Importantly, this staged release pattern effectively reduces the risk of eye or periorbital irritation caused by high local concentrations of the two drugs.

## 4. Discussion

The core-shell fiber membrane, engineered via coaxial electrospinning, integrates two distinct functional domains: a PLs-rich shell and an RSV-loaded core. This design addresses the critical limitations of conventional wound dressings, which often suffer from functional antagonism and uncontrolled release kinetics.

The outer shell of PLs causes membrane perforation through electrostatic attraction and physical disruption between the cationic groups and the anionic membrane. This ensures immediate antibacterial effects, especially against common Gram-positive bacteria and fungi in the eyes. In contrast, the RSV core uses its phenolic hydroxyl groups to eliminate ROS, thereby reducing oxidative stress and achieving slow drug release.

By separating the shell layer from the core solution using coaxial electrospinning technology, functional zones, controlled-release properties, and enhanced biocompatibility of core-shell nanofibers can be achieved. This structure prevents PLs from directly interacting with RSV, and the electrostatic-hydrophobic complementary interaction between the cationic shell layer and the hydrophobic core enhances the stability of the material in humid environments.

Although the two drugs showed differences in the initial release stage and release rate, and this difference was consistent with the expectation of the compartmental design. A more reasonable explanation is as follows: The drug in the outer shell layer is closer to the release interface and has a shorter diffusion path, which may result in faster surface dissolution and diffusion; while the drug in the core layer is subject to the combined regulation of the diffusion barrier of the outer shell and the coupled controlled-release effect of core hydration and expansion, as well as chain segment relaxation, and thus exhibits a relatively slower release behavior.

The PLs located in the PCL outer shell tend to achieve faster release in the early stage, thereby providing immediate antibacterial protection; while the RSV located in the PVA core is more likely to exhibit sustained release under the combined control-release effect of the outer shell barrier and core hydration-diffusion coupling, in order to maintain a longer period of antioxidant protection. The comprehensive research results indicate that the PCL-PLs/PVA-RSV core-shell nanofiber membranes can achieve “early antibacterial—subsequent antioxidant” staged functional output, which is in line with the usage scenario of the 8-h night sleep mask, and may reduce the risk of local short-term high concentration exposure-induced eye or periorbital irritation. To verify the performance of the proposed nanofiber membrane, we will modify the system to assess the morphological characteristics of the fibers, the stability of the core-shell structure, the kinetics of active component release, the antibacterial activity and antioxidant-related indicators, as well as the cell compatibility.

This fiber membrane can be applied to the cornea or conjunctiva, preventing postoperative infections and reducing oxidative damage to protect the corneal nerves. In the field of clinical translation, future technologies may integrate intelligent responsive materials to achieve on-demand drug release, transforming this material into an “active dressing” for dynamic ophthalmic treatment.

## 5. Conclusions

This study successfully employed coaxial electrospinning technology to fabricate a core-shell structure nanofiber membrane (PCL-PLs/PVA-RSV). Morphological characterization confirmed the formation of a unique coaxial structure, where PCL served as the protective shell and PVA-RSV was located at the core. The resulting membrane exhibited excellent hydrophilicity and high antibacterial activity, with a kill rate of over 98% against *E. coli* and *S. aureus*. Scanning electron microscope images showed that the presence of PLs caused significant damage to the bacterial cell membrane. Under the fluorescence microscope, the fibers showed a distinct “bright core–dark shell” fluorescence distribution, indicating that the drug was mainly loaded in the PVA core layer and the shell was completely covered. This was further confirmed by the side view of the transmission electron microscope, demonstrating the drug loading and structure construction of the core-shell type. Furthermore, the RSV-loaded membrane demonstrates potent antioxidant activity, achieving ABTS and DPPH radical scavenging rates of approximately 98%. The controlled release of the material’s core-shell structure demonstrated the release advantage of the fiber membrane, achieving programmed delivery of two drugs to the eye with a cumulative release rate of approximately 85%, ensuring efficient lesion repair. This asynchronous release strategy not only quickly establishes an ocular protective barrier but also provides long-term protection through a smooth sustained-release process, making it an ideal multifunctional drug delivery carrier for the eye.

Unlike traditional disposable eye patches, this study proposed a “functional replaceable core” design concept. As the core component of the eye mask, the nanofiber membrane achieved protection of the eye area through the antibacterial effect of the PCL-PLs outer layer and the antioxidant function of the PVA-RSV core. The existing results indicate that this core-shell structure design successfully integrated the biological activity of PLs and RSV onto a single platform. The absence of characteristic XRD peaks of PLs indicates that it achieved molecular-level dispersion in the fiber matrix, which is the source of the observed therapeutic effect. Although the experiments were conducted under standard laboratory conditions, their antibacterial and antioxidant properties, along with good biocompatibility and biocompatibility, provide a preliminary theoretical basis for the potential application of such functionalized membranes in multifunctional eye care materials.

## Data Availability

The original contributions presented in this study are included in the article. Further inquiries can be directed to the corresponding author.
